# Cost-effective adsorption of cationic dyes using ZnO nanorods supported by orange peel-derived carbon

**DOI:** 10.1038/s41598-025-86209-2

**Published:** 2025-02-03

**Authors:** Eman J. E. Abdelrazek, Ahmed A. Gahlan, Gamal A. Gouda, Abdelaal S. A. Ahmed

**Affiliations:** Chemistry Department, Faculty of Science, Al-Azhar University, Assiut, 71524 Egypt

**Keywords:** Recycling biowaste materials, Cationic dyes, Porous carbon, Adsorption, Pollution remediation, Chemistry, Environmental chemistry, Inorganic chemistry, Materials chemistry, Surface chemistry

## Abstract

Here, porous carbon (PC) and ZnO nanorods@PC (ZnO-NR@PC) composite derived from orange peel (OP) have been synthesized via a simple carbonization process. The prepared materials have been characterized by XRD, FT-IR, TEM, and BET analysis. The adsorptive properties of the prepared PC and ZnO-NR@PC composite have been investigated toward methylene blue (MB) and crystal violet (CV) cationic dyes from their aqueous solutions. The adsorption studies concluded that the maximum adsorption efficiency was achieved after 90 min in the basic conditions (pH = 10). Langmuir, Freundlich, Dubinin–Radushkevich (D-R), and Temkin non-linear isotherm models were applied to fit the experimental data. The adsorption of MB and CV dyes by the OP is fitted with the Freundlich model, and the adsorption of both dyes by the PC and the ZnO-NR@PC composite fitted with the Langmuir model. The estimated maximum adsorption capacity estimated from the adsorption of MB and CV by the ZnO-NR@PC composite was 74.45 and 74.89 mg/g, respectively. The calculated adsorption free energy from D-R and Temkin models indicates the adsorption of MB, and CV dye molecules by the OP, PC, and ZnO-NR@PC composite may be physical. The kinetic studies revealed the adsorption of MB and CV dyes onto the OP, PC and ZnO-NR@PC composite fitted with the pseudo-second-order model. On the otherhand, the thermodynamic studies confirmed the adsorption of MB, and CV dyes onto ZnO-NR@PC composite is an endothermic and spontaneous process. Furthermore, the prepared materials displayed high adsorption stability with an overall removal efficiency of about 90% after five cycles. The mechanism of MB and CV dyes by the ZnO-NR@PC composite is proposed to be controlled by electrostatic bonding, π-π interactions, and ion exchange. The results indicated the potential ability of OP-derived porous carbons as adsorbents for cationic dyes from aqueous media.

## Introduction

The rapid increase of the global population and the currently climatic changes lead to an increase in water pollution, thus increasing the freshwater crisis worldwide^1^. One of the major sources of water pollution is the recent rapid growth of industrial processes that depend on organic dyes. This industry leads to significant volumes of effluent from dyeing being discharged into the environment without sufficient treatment^2^. The dyes are highly poisonous and toxic due to the presence of aromatic rings and azo groups with the ability to produce toxic amines^3^. Therefore, these organic dyes pose a serious hazard to human health and aquatic ecology. One of the major issues caused by dyes in wastewater is that they block sunlight, which causes eutrophication and the death of aquatic life. Therefore, controlling or removing these organic dyes from wastewater before release into the environment is highly recommended. Several methods have been developed to eliminate the organic dyes from wastewater, such as membrane filtration^4^, photocatalysis^5–7^, sonophotocatalytic degradation^8^, ozonation^9^, advanced oxidation process (AOP)^10^, and chemical precipitation^11^. Among these techniques, the adsorption process has received great attention in various applications, including wastewater treatment, due to its simplicity, low cost, and relatively high efficiency^12,13^. In adsorption, the adsorbent material plays a critical role in the overall process; thus, it is important to find efficient adsorbent material^14^. Over the past decades, various adsorbents have been developed for dye removal, such as carbon-based materials^15,16^, metal-organic frameworks (MOF)^17^, metal oxides^5,18^, magnetic-based materials^19^, and polymers^20^. However, most of these materials require toxic and costly chemicals, which will affect the overall process. Therefore, developing low-cost, highly effective adsorbents is highly recommended for practical applications.

One of the promising materials is the agricultural biowastes, which displayed a potential ability as eco-friendly and low-cost adsorbent materials in wastewater treatment^21–24^. However, agricultural biowaste materials, without modification toward dye adsorption, are sometimes limited due to their lower efficiency^25^. Thus, great efforts have been devoted to converting such materials into another valuable adsorbent. Carbonization is a promising strategy for producing carbon materials with high porosity and surface area, which are critical for the overall adsorption efficiency. For example, Hegde et al.^26^, prepared carbon nanosphere from Arachis hypogaea for the removal of MB and malachite green (MG) cationic dyes from aqueous solutions. The removed efficiency reached 98% during 2 min with a maximum adsorption capacity of 1128.46 and 387.6 mg/g for MB and MG, respectively. Recently, L. Long et al. prepared nitrogen-doped cork-activated carbon (NCAC) by activation with melamine and K2CO3. The prepared NCAC displayed high adsorption of RhB dye with a maximum adsorption capacity of 2552.52 mg/g at 180 min. Another strategy is dispersing nanometal oxides as active centers into the porous carbon to enhance the efficiency of the adsorption process^27^. There are various metal oxides that have been utilized and dispersed in a carbon framework to be utilized for the elimination of organic pollutants from water. For example, Ahmed et al. dispersed NiO nanoparticles into the porous carbon derived from Thebaica (HT) seeds^28^. The adsorption of MR by the prepared material fitted with the pseudo-second-order kinetic and Langmuir isotherm models with an estimated maximum adsorption capacity of 129.87 mg/g. Additionally, ZnO is a famous metal oxide widely used for the removal of various organic pollutants from wastewater^18^. Gharaghani et al.^29^ prepared ZnO nanoparticles on glass plates for elimination of *p*-nitroaniline via photocatalytic degradation. The highest degredation efficiency was achieved at a pH of 7. Furthermore, the same group used a ZnO catalyst immobilized on a stone surface for degradation of acid red 18 (AR18) dye via a hybrid UV/COP advanced oxidation process with a maximum removal of 97%^30^. Recently, Daraei et al.^31^ used AgCuFe2O4@MWCNT/ZnO catalyts to eliminate the ceftriaxone (CFT) by photocatalytic degradation with a removal efficiency of 90.1%.

Furthermore, ZnO/and its composites are used as adsorbent materials; for example, recently, ZnO nanoparticles dispersed into activated carbon derived from wood sawdust (ZnO@AC) were prepared as adsorbents for anionic dyes^27^. Here, we prepared porous carbon (PC) and ZnO nanorods (ZnO-NR) impregnated into the PC derived from OP as adsorbents for MB and CV dyes. The prepared were used to eliminate the cationic dyes from aqueous media. The influence of pH, contact time, adsorbent doses, and initial dye concentrations on the overall removal performance was investigated. Furthermore, the adsorption kinetics, adsorption isotherm, and thermodynamics were studied.

## Experimental section

### Materials

In this study, all chemicals were of analytical grade. Sodium hydroxide (NaOH, ≥ 98%, pellets anhydrous), hydrocholoric acid (HCl, 37%), zinc acetate dihydrate (Zn(CH_3_COO)_2_·2H_2_O, ≥ 98%), and absolute alcohol (C_2_H_5_·OH, ≥ 95%) were purchased from Merck, Darmstadt, Germany. Crystal violet (C_25_H_30_ClN_3_; M.wt. 407.99 g/mol) and methylene blue (C_16_H_18_ClN_3_S; M.wt. 319.85 g/mol) were from Alpha Chemika, India. All used reagents were of analytical purity and used as received. De-ionized (DI) water was obtained from an ultra-pure purifier (Ulupure, resistivity ≥ 18.2 MΩ).

### Preparation of adsorbent materials from waste orange peel (OP)

**Fig. 1:**
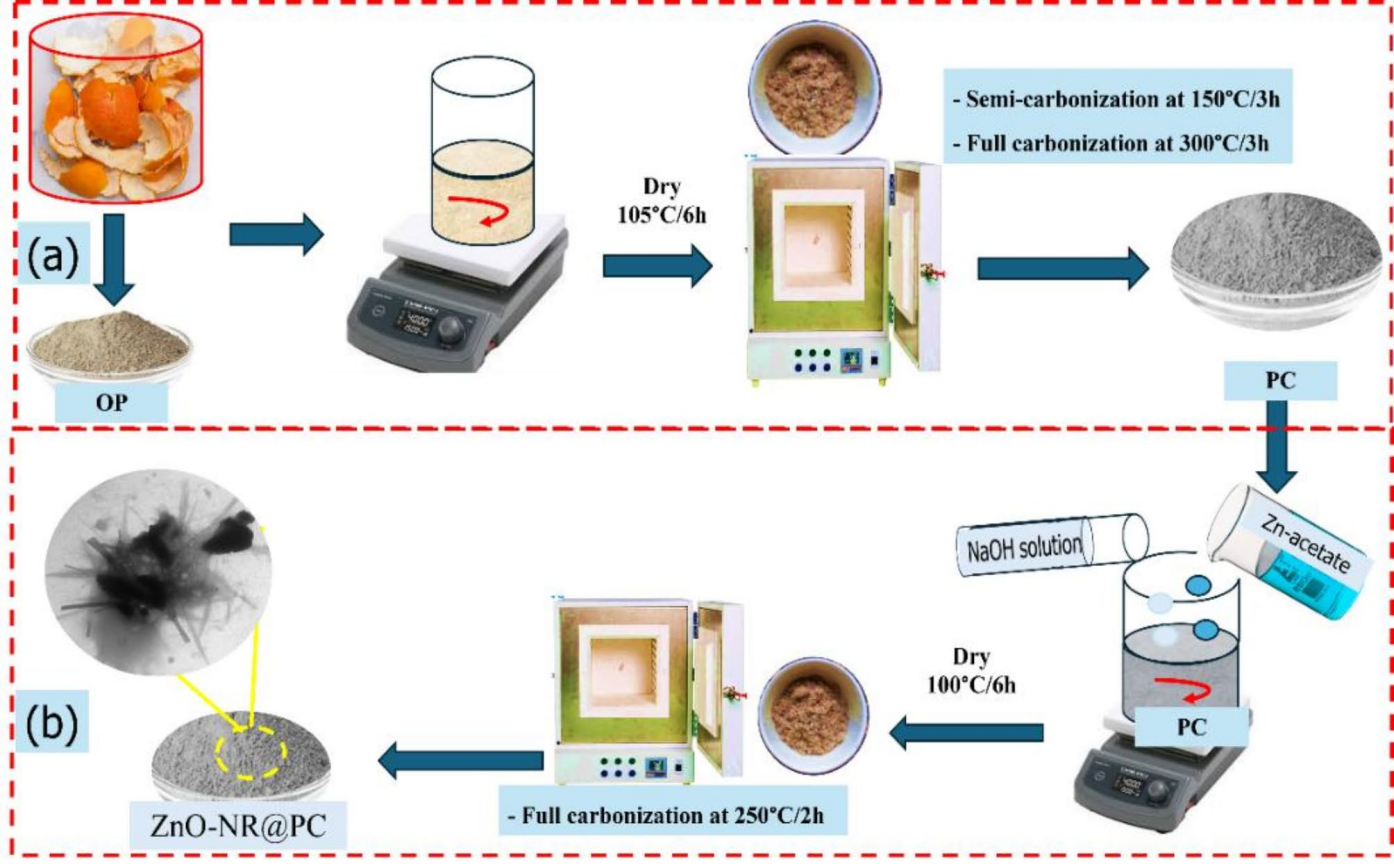
Scheme for preparation of the (**a**) PC and (**b**) ZnO-NR@PC composite derived from waste OP.

In our study, the orange fruit was obtained from the local market in Assiut governorate, Egypt. Two adsorbent materials derived from waste orange peel (OP); these materials are known as porous carbon (PC) and zinc oxide nanorods dispersed in porous carbon (ZnO-NR@PC) composite as described in Fig. 1. For the preparation of PC in Fig. 1a, the waste OP was washed thoroughly with tap water to remove any dust and impurities. Then washed with hot tap water to remove the color, followed by washing three times with distillated water, and then subjected to drying for 6 h at 105 °C. The dried sample was subjected to semi-carbonization for 3 h at 150 °C and finally subjected to full carbonization for 3 h at 300 °C. The obtained black powder was washed by dist. H_2_O and dried at 110 °C, and the obtained sample is marked as PC. For the ZnO-NR@PC composite (Fig. 1b), exactly 0.5 g of the PC derived from waste OP was mixed with 50 mL of an aqueous solution of 0.25 M Zn-acetate under a strong magnetic stirrer at 60 °C for 1 h. After that, a 0.5 M NaOH solution was dropped onto the above mixture and kept under a magnetic stirrer at 60 °C for a further 2 h. The solid product was collected by centrifugation at 3000 rpm and washed thoroughly with distilated water till pH reached ≈ 7 and washed once with ethanol. Finally, the solid product dried at 100 °C overnight and was subjected to carbonization at 150 °C for 2 h. The obtained material is called ZnO-NR@PC composite.

### Characterization

The prepared PC and ZnO-NR@PC composite derived from OP were characterized by various techniques. The functional groups were studied by Fourier transform infrared spectroscopy (FT-IR). The FTIR spectrum was recorded in the 4000–400 cm^−1^ regions at a resolution of 2 cm^−1^ on a Nicolet spectrophotometer (model 6700) by using the KBr pellet technique. The crystallinity of the materials was investigated by X-ray diffraction (XRD) using a Bruker D8 advanced X-ray diffractometer (monochromatic Cu-Kα radiation source, λ = 0.15406 nm) running at 40 kV. The specific surface area and the pore size distribution of the prepared samples were determined using the Brunauer-Emmett-Teller (BET) and Barrett-Joyner-Halenda (BJH) models by the N_2_ adsorption-desorption technique on a Micromeritics ASAP 2020 HD88 system. Transmission electron microscopy (TEM) images were obtained by a JEM-2100 F field emission microscope (JEOL Ltd., Japan) with an accelerating voltage of 200 kV used to investigate the surface morphology of the prepared materials. The point of zero charge (pH_PZC_) of the prepared materials was determined by the drift method. Six samples of 20 mL of 0.1 M NaNO_3_ solutions with initial pH_i_ values ranging from 2 to 12 were prepared and adjusted by 0.1 M HCl and 0.1 M NaOH solutions^32^. Then, 0.03 g of absorbent was added to each solution and kept under shaking at room temperature for 24 h. The final pH_f_ values of the supernatant liquid were determined by plotting pHi vs. pH_f_, the point of zero charge (pH_PZC_).

### Adsorption study

In our work, three materials known as OP, PC, and ZnO-NR@PC composite were prepared from waste OP as adsorbents toward MB and CV cationic dyes from aqueous solution. In each experiment, 25 mL of each dye solution was mixed with 0.05 g of adsorbent and kept under a magnetic stirrer at a constant speed of 300 rpm for the desired time. The effect of solution pH (2.5–12.5), contact time (5–120 min), initial dye concentration (10–200 mg/L), adsorbent dosage (10–100 mg/25 ml), and temperature (25–70 °C) on the overall removal efficiency (R%) was studied. The pH values of dye solutions were adjusted by 0.1 M HCl and 0.1 M NaOH aqueous solutions. After the adsorption process at desired conditions, the remaining MB and CV dye concentrations were determined, respectively, at 664 and 590 nm, by UV-visible spectrophotometer. The removal efficiency (R%) was determined by Eq. (1), and the maximum adsorption capacity (q_e_; mg/g) of the adsorbent materials was calculated by Eq. (2).

1 $${\text{R}}\;~(\% )=\frac{{{{\text{C}}_0} - {{\text{C}}_e}}}{{{{\text{C}}_0}}}~ \times ~100$$

2 $${{\text{q}}_{\text{e}}}=\frac{{({{\text{C}}_0} - {{\text{C}}_{\text{e}}})}}{{\text{M}}}~~{\text{V}}$$

Where C_0_ and C_e_ are the initial and final dye concentrations (mg/L), respectively. V (L) is the volume of dye solution, and M (g) is the mass of the adsorbent.

## Results and discussion

### Characterization of adsorbents derived from waste OP

**Fig. 2:**
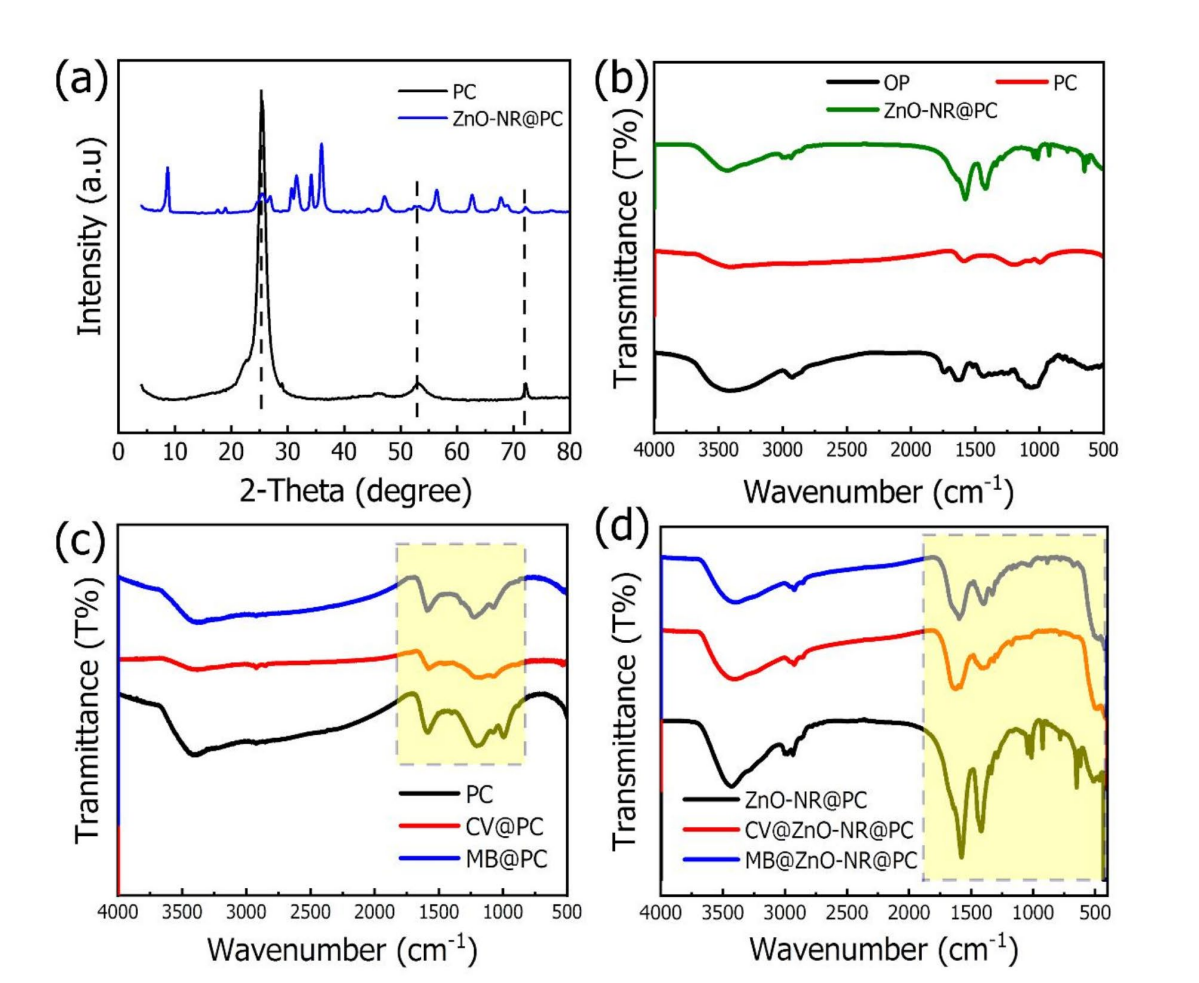
(**a**) XRD patterns of PC and ZnO-NR@PC composite derived from OP, and (**b**–**d**) FT-IR spectra of PC and ZnO-NR@PC composite with and without absorbed MB and CV dyes.

Figure 2a displays the XRD patterns of the PC and ZnO-NR@PC composite. The pattern of the PC exhibited two peaks located at 2θ = 25.5°, 54.5°, and 73.2°. The first two peaks are respectively attributed to the (002) and (100) planes of graphite, which indicates the full conversion of OP to porous carbon^33^. The diffraction patterns of the ZnO-NR@PC composite are properly indexed to hexagonal wurtzite ZnO (JCPDS 36-1451) and match well with the previously published literature^34,35^. This indicates indicates successfully PC and ZnO-NR@PC composite from OP.

The FTIR spectra of the prepared materials with and without adsorbed dyes are displayed in Fig. 2b–d. The FT-IR spectra of the prepared materials before dye adsorption in Fig. 2b displayed broad beaks located at 3500 cm^−1^ for the bands of the O-H groups from the vibration of absorbed water molecules. In addition, the OP spectrum displayed a weak peak located at 2945 cm^−1^ for the C–H (CH_2_) bond stretch vibration. Also, two peaks were observed in 1750 and 1620 cm^−1^ which are respectively assigned to the C=O and C=C groups. The broad peak located at 1000–1100 cm^−1^ is due to the presence of the C–O group in the OP sample^36^. For the ZnO-NR@PC composite spectrum, there are small bands between 650 and 1000 cm^−1^ assigned for Zn-O. The FTIR spectra of PC loaded with the MB and CV dyes presented in Fig. 2c. It can be observed that the spectra of PC before and after MB dye adsorption are the same, indicating no chemical reactions occur, and this supports the physical adsorption process between PC and MB dye molecules. On the other hand, after CV adsorption, the intensities of all peaks are reduced, which can be attributed to some chemical bonding between PC and CV dye molecules, which support the chemisorption process. Figure 2d displayed the FT-IR spectrum for ZnO-NR@PC composite with and without MB and CV dye adsorption. The peaks located between 4000 and 2000 cm^−1^ are the same before and after adsorption. However, the peaks located in the yellow box are noticeably changed, which can be assigned to the chemical bonding between ZnO with the dye molecules. This confirms a chemical adsorption process between ZnO-NR@MB and CV dye molecules.

**Fig. 3:**
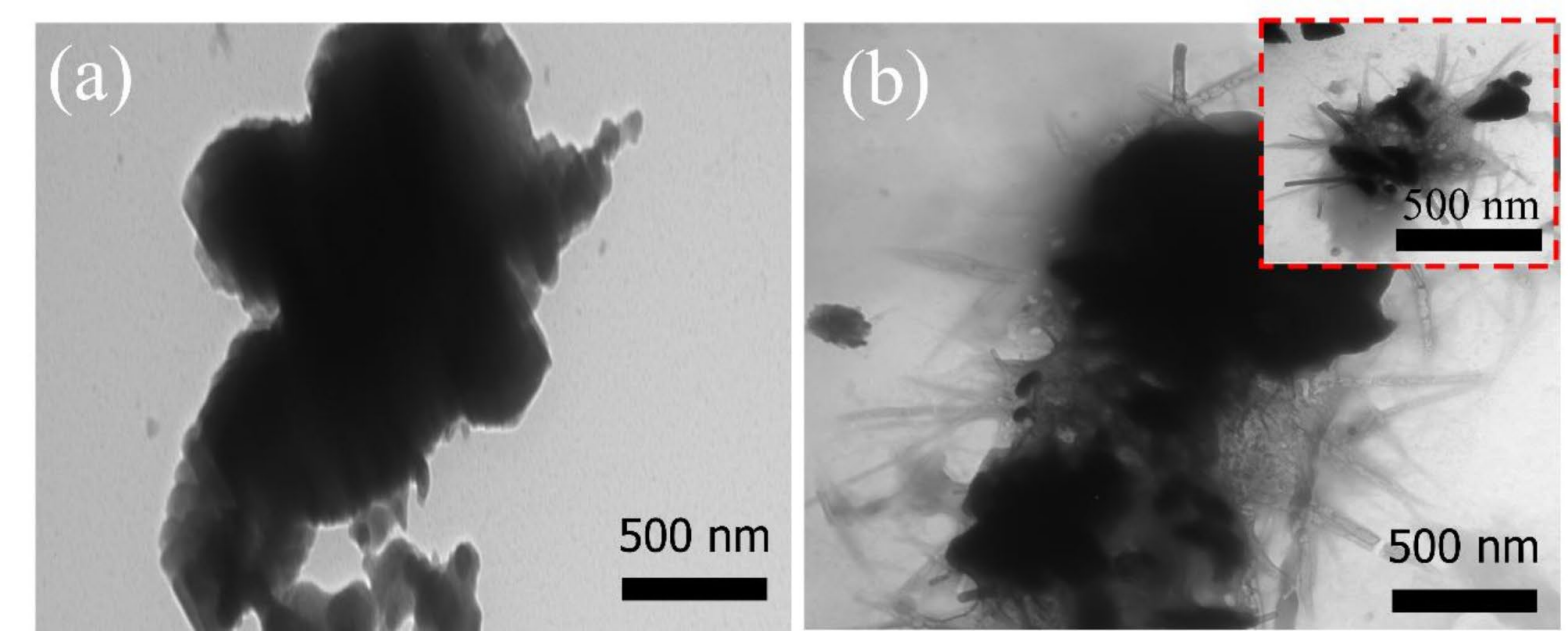
TEM images of (**a**) PC and (**b**) ZnO-NR@PC composite derived from waste OP.

To investigate the surface features of the prepared PC and ZnO-NR@PC composite, TEM techniques were utilized. From Fig. 3a, the TEM image of the prepared PC displayed porous carbon sheets, while the ZnO-NR@PC composite (Fig. 3b) showed the formation of ZnO nanorods (ZnO-NR) surrounded by the porous carbon sheets. Combined with the XRD results, we confirmed the successful preparation of the PC and ZnO-NR@PC composite.

**Fig. 4:**
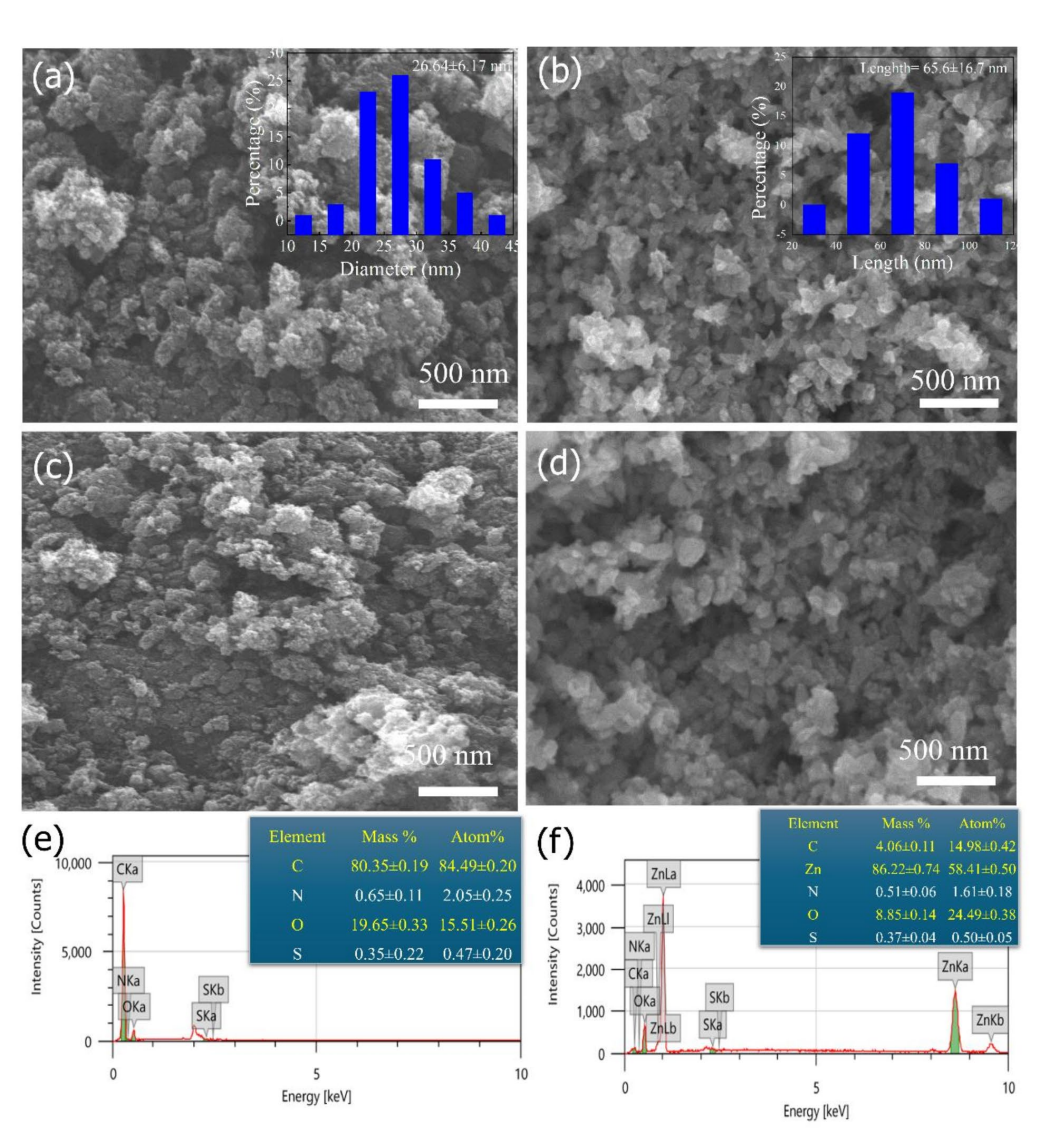
SEM images of the prepared PC (**a**) and ZnO-NR@PC composite (**b**). SEM and EDX analyses of PC (**c**,**e**) and ZnO-NR@PC composite (**d**,**f**) after MB dye adsorption.

The surface changes in the prepared adsorbent materials before and after the adsorption process were studied by SEM and energy-dispersive X-ray spectroscopy (EDX). The SEM image of the PC in Fig. 4a displayed highly porous materials with an average particle size of 26.64 ± 6.2 nm. On the other hand, the SEM image of the ZnO-NR@PC composite (Fig. 4b) showed a presence of ZnO nanorodes with average lengths of 65.6 ± 16.7 nm and average diameters of 35.30 ± 7.31 nm. After MB dye adsorption, both PC (Fig. 4c) and ZnO-NR@PC (Fig. 4d) displayed some aggregations due to the presence of MB dye in the adsorbent layers and pores. This confirms the ability to remove MB dye from an aqueous solution. Also, EDX analysis (Fig. 4e,f) confirm the presence of N and S ions in both PC and ZnO-NR@PC, which indicates the presence of MB dye molecules after adsorption.

**Fig. 5:**
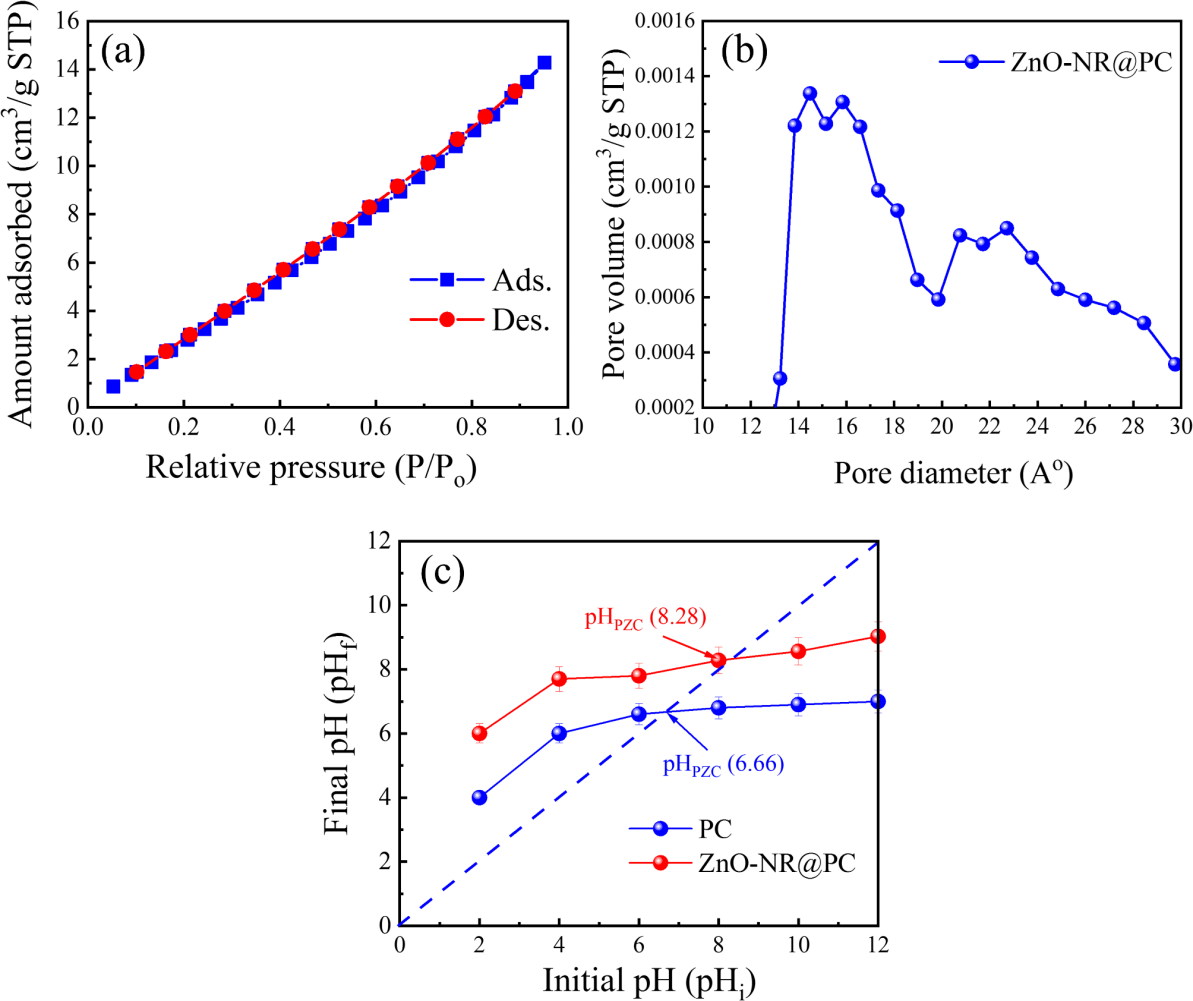
(**a**) Nitrogen adsorption-desorption isotherms and (**b**) pore size distribution of ZnO-NR@PC. (**c**) The pH_PZC_ of the PC and ZnO-NR@PC composite.

The specific surface area plays a critical role in the overall adsorptive properties. Thus, the N_2_ adsorption-desorption isotherm technique was utilized to investigate the specific surface area (S_BET_) and pore size distribution of the ZnO-NR@PC composite. As presented in Fig. 5a, the obtained N_2_ adsorption isotherms belong to type IV with an H_3_ hysteresis loop, which is typical of mesoporous materials according to the IUPAC classification^37^. The S_BET_ surface area of the ZnO-NR@PC composite was estimated to be 19.12 m^2^/g, and the pore volume is 2.21 cm^3^/g. A Barrett-Joyner-Halenda (BJH) analysis was used to determine the distribution of pore sizes (Fig. 5b), which showed the presence of macropores and mesopores in the sample^38^.

The point of zero charge (pH_PZC_) is an important factor to determine the surface charge of the adsorbent^39^. Therefore, pH_PZC_ is used to show the attraction and repulsion forces between the absorbent and adsorbate during the adsorption process^40^. Here, we determine the pH_PZC_ by the pH drift method^32^. As in Fig. 5c, the pH_PZC_ values are 6.66 and 8.28 for the PC and the ZnO-NR@PC composite, respectively. At pH > pH_ZPC_, the adsorbent surface has negative charges, and at a pH < pH_ZPC_, the adsorbent surface has positive charges^41^.

### Adsorption studies

#### Effect of pH

**Fig. 6:**
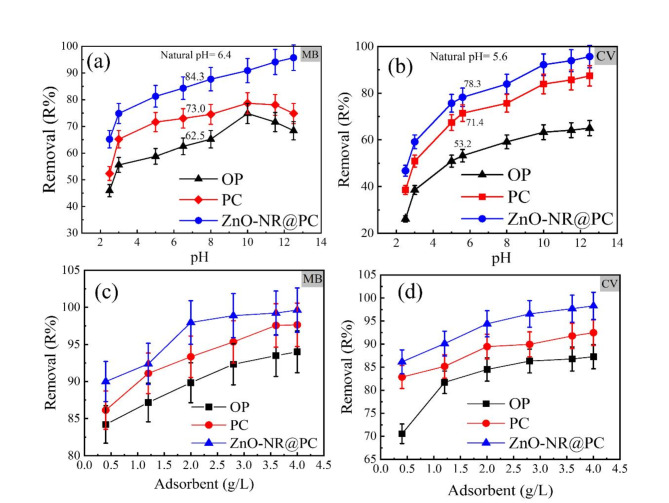
Effect of (**a**,**b**) solution pH [C_0_ = 50 mg/L, M = 0.05 g, t = 60 min, pH 2.5–12.5] on the removal of MB and CV dyes from their aqueous solution. (**c**,**d**) Effect of adsorbent dosage on the removal of (**c**) MB and (**d**) CV dyes from their aqueous solutions [M = 0.01–0.1 g, t = 90 min C_0_ = 50 mg/L, pH 10].

The initial pH of the adsorbent solution plays a critical role in the overall adsorption process due to its strongly effect on the surface charge of the adsorbent and adsorbate molecules^42^. Furthermore, the functional groups on the adsorbent or adsorbate are protonated and deprotonated by adjusting the pH value; thus, an electrostatic contact between the adsorbent and adsorbate molecules may occur. It has been reported that both the pKa of the adsorbate and the pHpzc of the adsorbent play a critical role in the adsorption process^43^. For MB dye, the reported pKa values for MB and CV are 3.8 5.31, respectively. Thus, at a higher pH value (pH > pKa), the MB and CV species in solution are cationic. Additionally, at pH > pH_ZPC_, the surface of the adsorbent is negative, which promotes the electrostatic attraction and thus enhances the adsorption efficiency. In this regard, the effect of pH on the adsorption of MB and CV dyes by the OP, PC, and ZnO-NR@PC composite was investigated. As in Fig. 6a,b, the highest removal percentages of MB and CV dyes were achieved and at a pH of 10 (pH > pH_PZC_). For the adsorption of MB dye by the OP, PC, and ZnO-NR@PC composite, the removal reached 74.8, 78.70, and 90.91%, respectively. On the other hand, the removal percentages of CV dye reached 63.28, 83.90, and 92.16% by the OP, the PC, and the ZnO-NR@PC composite, respectively. From Fig. 6a,b, the removal of both dyes at pH 10 is much higher than those achieved by natural dye solutions at pH 6.38 (MB) and 5.58 (CV), which indicate the necessity of adjusting the pH of solution to maximize the adsorption efficiency. This is in accordance with the previous discussion, as at the pH < pH_PZC_, the surface of adsorbents is positive and repelled the cationic dye molecules, causing low removal efficiency. Additionally, by increasing the pH values (pH < pH_PZC_), there was an increase in the surface negative charges, enhancing the electrostatic attraction of the cationic dye molecules. Therefore, electrostatic interaction was a major mechanism in the adsorption of cationic dyes (MB and CV) on the OP, PC, and ZnO-NR@PC.

#### Effect of adsorbent dosage

The effect of adsorbent dosages on the removal efficiency of MB and CV dyes was investigated, and the obtained data are presented in Fig. 6c,d. The removal percentage gradually increased as the adsorbent doses increased from 0.4 to 4 g/L. The higher adsorbent dosage indicates higher active sites available for the interaction with the dye molecules, which raises the overall removal efficiency.

#### Effect of dye initial concentration and isotherm study

**Fig. 7:**
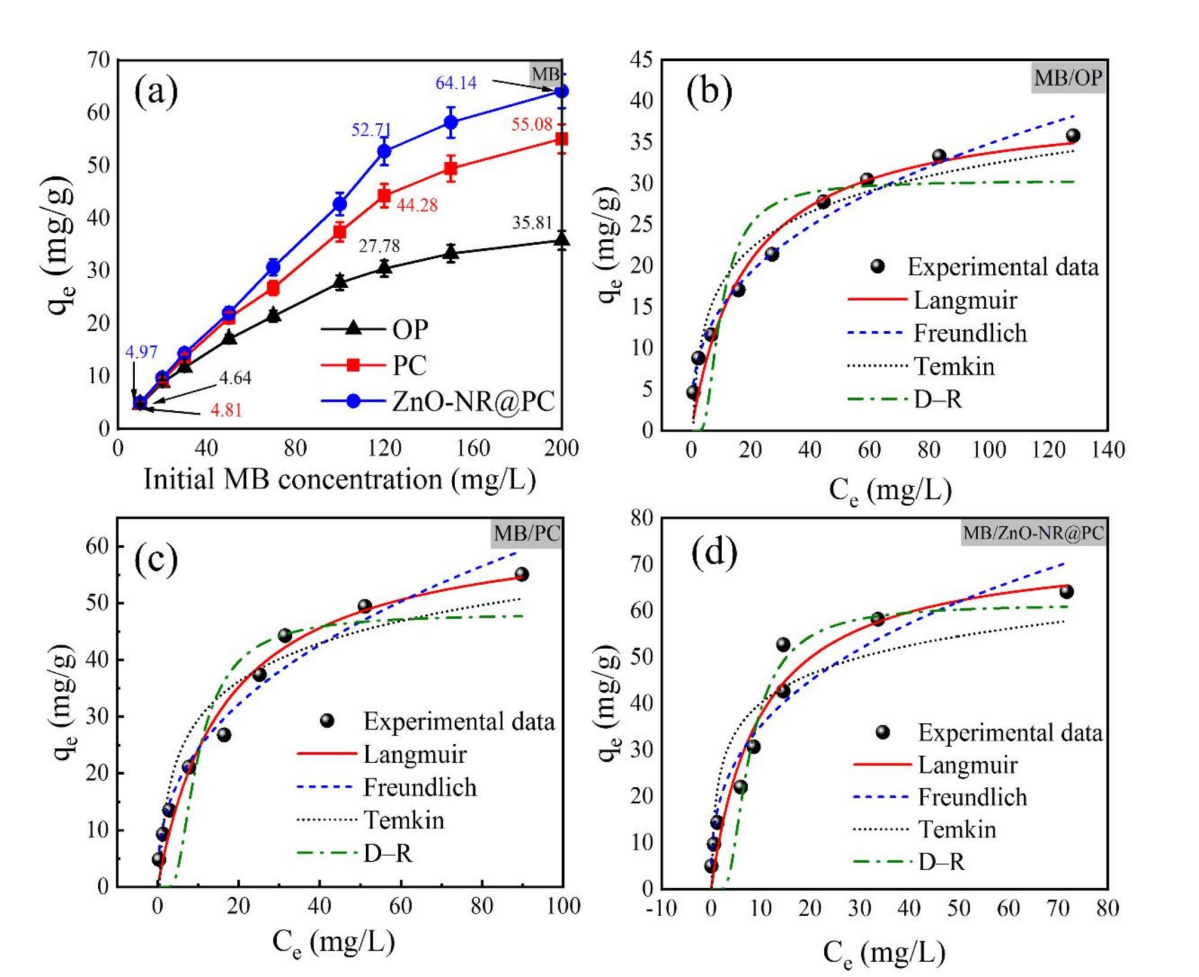
The experimental and adsorption isotherm of MB onto the OP, PC, and ZnO-NR@PC composite [C_0_ = 10–200 mg/L, M = 0.05 g, pH 10, time = 90 min].

**Fig. 8:**
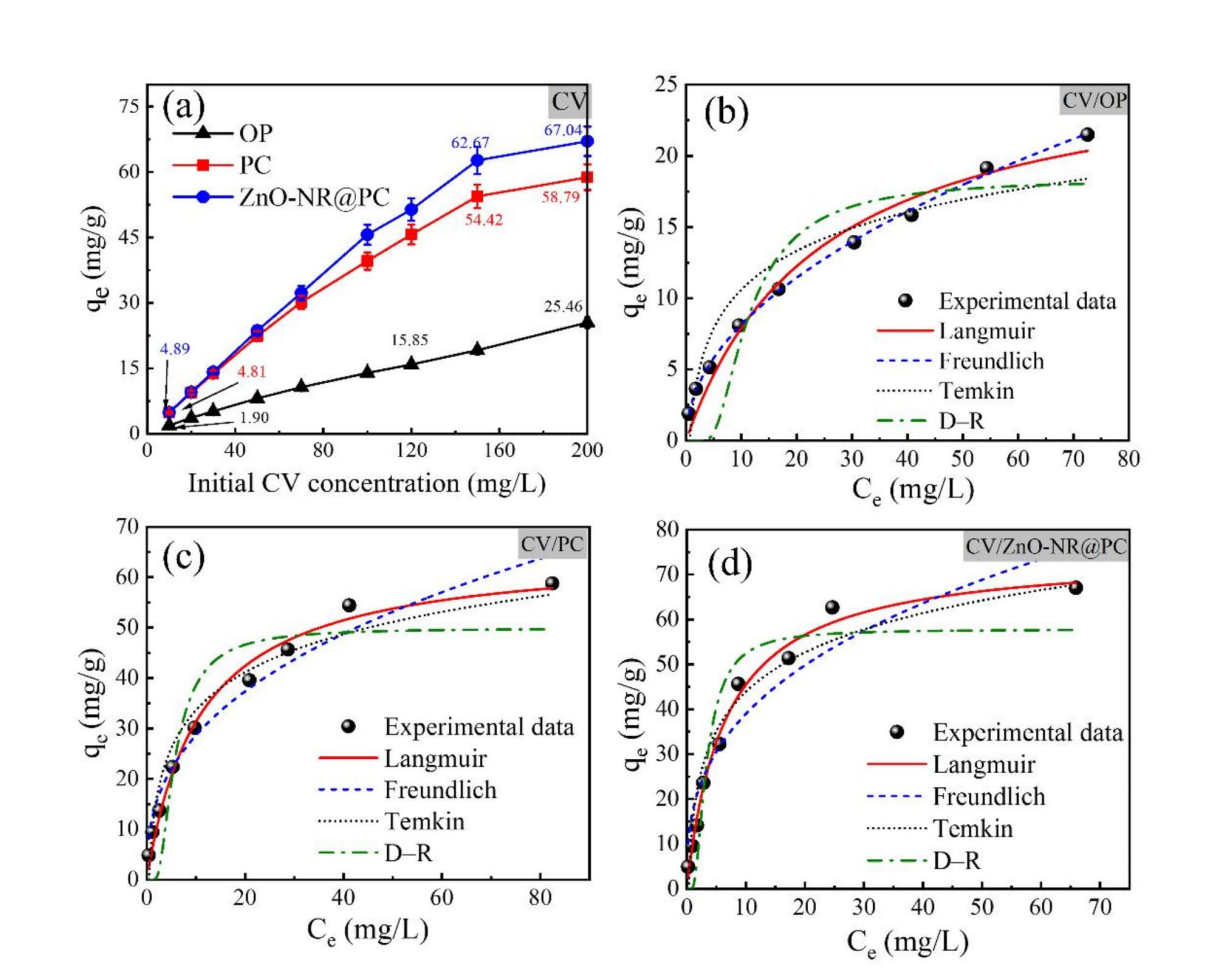
The experimental and adsorption isotherm of adsorption CV onto the OP, PC, and ZnO-NR@PC composite [C_0_ = 10–200 mg/L, M = 0.05 g, pH 10, time = 90 min].

The effect of initial MB and CV dye concentrations on the adsorption capacity was investigated in the range of 10–200 mg/L. From Fig. 7a, the adsorption of MB dye by the OP displayed the lowest adsorption capacity, and the highest adsorption capacity was achieved by the ZnO-NR@PC composite. By increasing the initial concentration of MB dye from 10 to 120 mg/L, the adsorption capacity of OP is increased from 4.64 to 30.44 mg/g. The obtained adsorption capacity of the PC and the ZnO-NP@PC composite are increased from 4.82 to 44.27 mg/g, and from 4.97 to 52.72 mg/g respectively. The same behaviour was observed for the adsorption of CV dye as in Fig. 8b. The adsorption capacity increased from 1.90 to 15.58 mg/g for the OP, from 4.81 to 54.42 mg/g for the PC, and from 4.89 to 62.67 mg/g for the ZnO-NR@PC composite. The main reason for enhancing adsorption capacity by increasing the initial dye concentration is due to the improvement in the mass transfer driving force via several collisions between dye molecules and the surface of the adsorbent. The slight enhancement of adsorption capacity at higher concentrations higher than 120 mg/L can be assigned to the growing repulsion forces between the bulk phase and dye molecules on the surface of the adsorbent after initial adsorption^44^. In order to investigate the nature of the adsorption and the equilibrium behavior between the adsorbent-adsorbate molecules, four adsorption isotherm models in non-linear forms (Langmuir, Freundlich, Dubinin–Radushkevich (D-R), and Temkin) were applied to fit the experimental data^45^. The non-linear plots are in Figs. 7c,d and 8c,d and the related parameters are in Table 1.

The Langmuir model assumes that the adsorbent surface is homogeneous with a finite number of identical sites; thus, a monolayer of adsorbate is chemically attached to the surface of the adsorbent. The non-linear form of the Langmuir isotherm can be expressed by Eq. (3)^46^.

3 $${q_e}\frac{{{q_{max}}{K_L}{C_e}}}{{1+{K_L}{C_e}}}$$

Where C_e_ (mg/L) is the equilibrium concentration, q_max_ (mg/g) is the maximum adsorption capacity, and K_L_ (L/mg) is the separation factor related to the adsorption affinity of the binding sites. The R_L_ value indicates whether it is linear (K_L_ = 1), irreversible (K_L_ = 0), favorable (0 < K_L_ < 1), or unfavorable (K_L_ > 1).

The Freundlich model is used to describe the heterogeneous surfaces, in which its non-linear form can be expressed by Eq. (4).

4 $${q_e}={K_f}C_{e}^{{1/n}}$$

Where *K*_f_ (L/g) is constant represents the adsorption capacity. The adsorption power (1/n) is represent the adsorption intensity and used to show the ideality and limit of the adsorbent/adsorbate interaction.

Depending on the higher R^2^ and the lower χ^2^ values in Table 1, we can summarize the observations as follows: (i) the adsorption of MB and CV dyes by the OP is fitted well with the Freundlich isotherm model, and (ii) the adsorption of MB and CV dyes onto the PC and the ZnO-NR@PC composite obeyed the Langmuir isotherm model, confirming monolayers of dyes cover the surface of the adsorbent. The K_L_ values falling within the range of 0–1, which indicates favorable adsorption of the dyes on our prepared adsorbent materials^47^. In addition, the estimated K_L_ values for the adsorption of MB and CV dyes by the ZnO-NR@PC composite are higher than those obtained from the adsorption by the OP and PC materials, demonstrating more stable interactions between the MB and CV dye molecules with the ZnO-NR@PC composite. From the Langmuir model, the maximum adsorption capacity from the adsorption of MB by the OP, the PC, and ZnO-NR@PC composite was estimated to be 39.97, 64.72, and 74.45 mg/g, respectively. Moreover, the maximum adsorption capacity from the adsorption of CV dye by the OP, PC, and ZnO-NR@PC composite is 27.20, 65.47, and 74.89 mg/g, respectively. The estimated adsorption capacity showed a good match with the experimental values. In comparison with the pristine OP and the PC, the ZnO-NR@PC displayed higher adsorption capacity due to the presence of ZnO nanorods. From the Freundlich isotherm model, the adsorption intensity (1/*n*) all below one, which confirms the favorable adsorption of MB and CV dyes under working conditions. Morovore, the adsorption capacity (*K*_F_) is ordered as OP < PC < ZnO-NR@PC composite. The higher *K*_F_ for adsorption of dyes onto the ZnO-NR@PC composite indicates a faster adsorption rate. Although Langmuir and Freundlich are widely utilized to study the isotherms of the adsorption, they are not accurate in differentiating between the chemical and physical adsorption processes. Therefore, it is necessary to include Temkin and D-R isotherms to examine the adsorption process type^48^. The D-R model was used to predict the adsorption onto heterogeneous surfaces and to determine the mean free adsorption energy (E_DR_ = 1/√2β). The estimated E_DR_ value used indicates the adsorption mechanism, whether it is physical or chemical adsorption or an ion exchange process^49^. This non-linear form of the D-R model is expressed by Eq. (5).

5 $${q_e}={q_{DR}}\exp ( - \beta {\varepsilon ^2})$$

Where q_DR_ (mg/g) is the theoretical adsorption capacity, β (mol^2^/kJ^2^) is the D-R constant, ε is Polanyi potential; $\varepsilon ={\text{RT}}\;\ln ~\left( {1+1/{\text{Ce}}} \right).$ R is the universal gas constant (8.314 J/mol·K), and T (K) is the absolute temperature.

Based on the adsorption energy, the adsorption process is classified as chemical (8 < E_DR_ < 16 kJ/mol) or physical (E_DR_ < 8 kJ/mol). As presented in Table 1, the estimated E_DR_ values are less than 8 kJ/mol, which means that the adsorption processes are physical. The Temkin isotherm is a model used to describe the chemsorption process and predict the uniform distribution of binding energies over the population of surface binding adsorption^50^. The non-linear equation of the Temkin isotherm as Eqs. (6–8).

6 $${q_e}={B_T}\ln ({a_t}{C_e})$$

7 $${B_T}=\frac{{RT}}{{bt}}$$

8 $${q_e}=\frac{{RT}}{{{b_t}}}\ln \left( {{a_t}{C_e}} \right)$$

The a_t_ (L/g) is equilibrium binding constant, and b_t_ as Temkin isotherm constant.

From Table 1, the corresponding b_t_ values are in the range of 0.196 and 0.629 kJ/mol, all of which are lower than 8 kJ/mol. This indicates weak interactions between the MB and CV dye molecules and the surfaces of the OP, PC, and ZnO-NR@PC composite. Thus, the adsorption processes may be physical. In addition, the value a_t_ obtained form the adsorption of MB and CV by the ZnO-NR@PC composite are higher than those from the OP and PC, which indicate stronger binding.

**Table 1:** Isotherm parameters for adsorption of MB and CV dyes onto OP, PC, and ZnO-NR@PC composite.

Isotherm model	Parameters	OP	PC	ZnO-NR@PC
MB adsorption				
Langmuir	q_e_ mg/g	39.97	64.72	74.45
	K_L_ L/mg	0.054	0.060	0.102
	Adj. R^2^	0.959	0.975	0.937
	χ^2^	5.314	6.154	30.53
Freundlich	K_f_ L/g	0.384	9.536	15.68
	1/n	0.368	0.406	0.351
	Adj. R^2^	0.982	0.968	0.912
	χ^2^	2.278	10.591	42.802
Dubinin-Radushkevich	q_max_ (mg/g)	30.38	48.20	61.50
	β (mol^2^/kJ^2^)	80.99	81.57	50.8
	E (kJ/mol)	2.485	2.476	3.136
	Adj. R^2^	0.738	0.781	0.879
	χ^2^	33.50	73.27	58.67
Temkin	a_t_ (L/g)	1.624	2.074	8.818
	b_t_ (kJ/mol)	0.389	0.255	0.276
	Adj. R^2^	0.938	0.910	0.812
	χ^2^	7.954	30.150	91.366
CV adsorption				
Langmuir	q_e_ mg/g	27.20	65.47	74.89
	K_L_ L/mg	0.041	0.092	0.154
	Adj. R^2^	0.968	0.984	0.988
	χ^2^	1.516	6.154	6.223
Freundlich	K_f_ L/g	2.625	11.802	17.245
	1/n	0.492	0.385	0.354
	Adj. R^2^	0.999	0.963	0.899
	χ^2^	0.067	14.492	54.036
Dubinin-Radushkevich	q_max_ (mg/g)	18.40	49.90	57.79
	β (mol^2^/kJ^2^)	104.33	28.46	10.78
	E (kJ/mol)	2.189	4.191	6.810
	Adj. R^2^	0.789	0.825	0.876
	χ^2^	10.22	68.46	66.46
Temkin	a_t_ (L/g)	1.480	2.188	3.217
	b_t_ (kJ/mol)	0.629	0.227	0.196
	Adj. R^2^	0.890	0.949	0.928
	χ^2^	5.319	19.814	38.601

#### Effect of contact time and adsorption kinetics

**Fig. 9:**
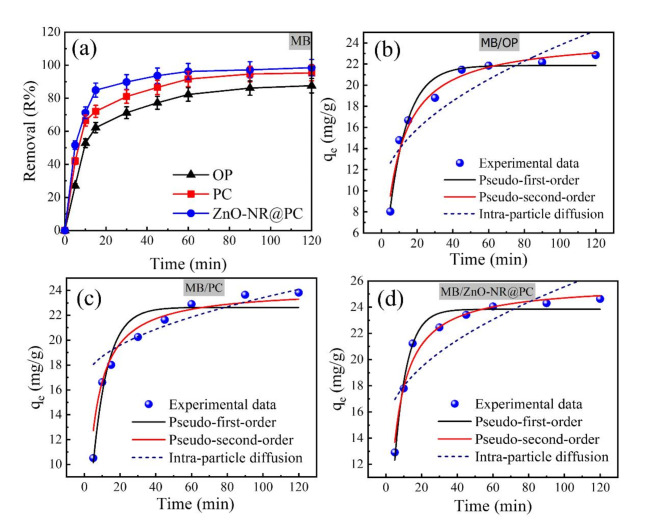
(**a**) Effect of contact time on the removal of MB dye from aqueous solution. (**b**–**d**) Experimental and kinetic adsorption models for the adsorption of MB onto OP, PC, and ZnO-NR@PC composite. [C_0_ = 50 mg/L, M = 0.05 g, pH 10, t = 5–120 min].

The effect of contact time on the adsorption efficiency was examined in the durations ranging from 5 to 120 min. The MB and CV dye solutions were agitated with 0.05 g of adsorbent material at room temperature. From Fig. 9a, by increasing the contact time from 5 to 90 min, the removal percentage of MB dye increased from 26.16 to 65.03% and from 38.53 to 87.41% by using OP and PC, respectively. The higher removal percentage is observed by the ZnO-NR@PC composite, as indicated by increasing the value from 46.78 to 95%.

**Fig. 10:**
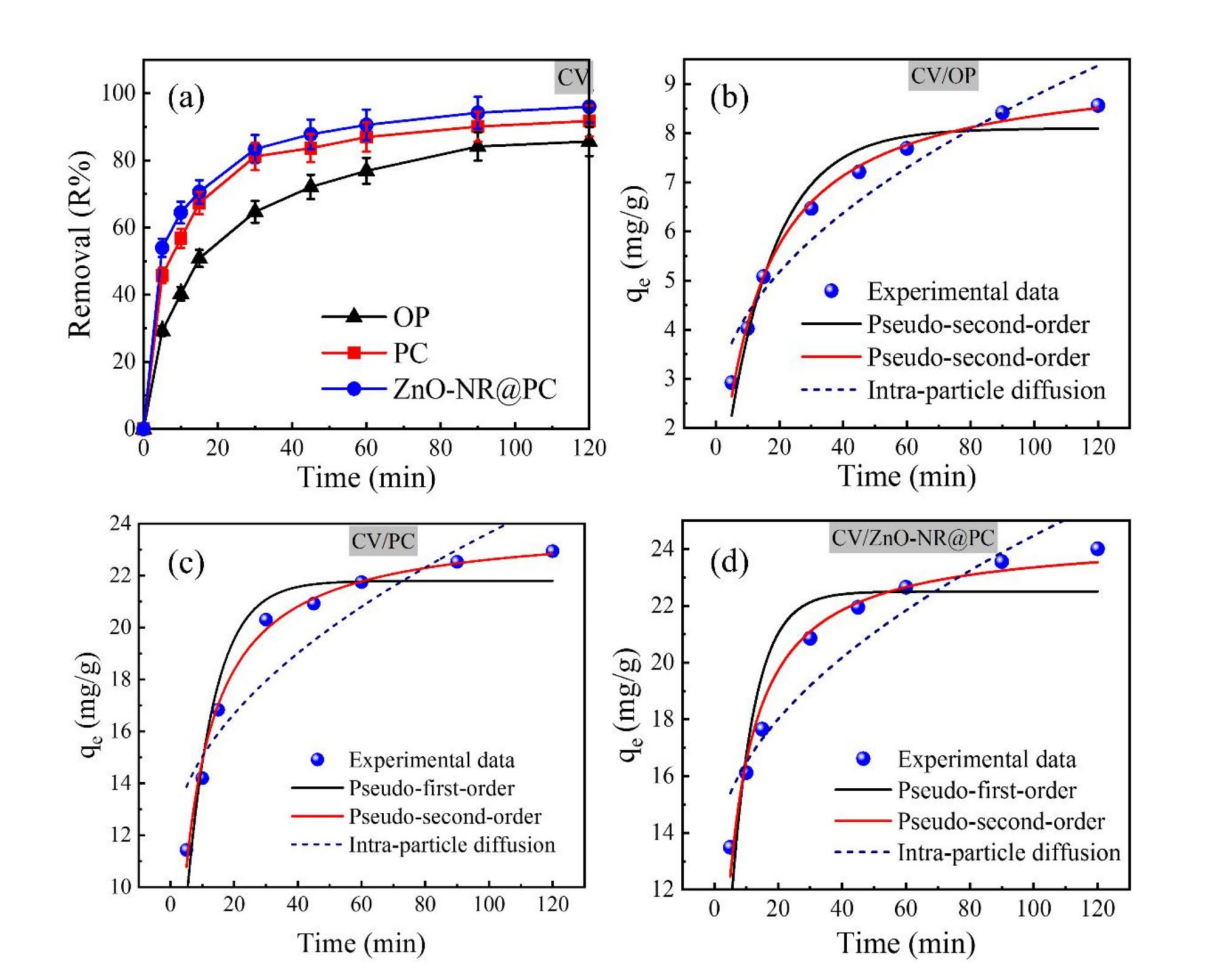
(**a**) Effect of contact time on the removal of CV dye from aqueous solution. (**b**–**d**) Experimental and kinetic adsorption models for the adsorption of CV onto OP, PC, and ZnO-NR@PC composite. [C_0_ = 50 mg/L, M = 0.05 g, pH = 10, t = 5–120 min].

As observed in Fig. 10a, the adsorption of CV dye by the our adsorbents exhibited the same behavior as MB adsorption. By increasing the contact time from 5 to 90 min, the removal percentage of CV dye increased from 29.22 to 85.66% and from 45.73 to 91.77% by the OP and PC, respectively. The higher percentage was achieved by the ZnO-NR@PC composite, as indicated by increasing from 53.98 to 96.04%. The remarkable increases in adsorption at the beginning can be attributed to the presence of large numbers of available active sites on the adsorbent surface. Higher contact time (> 90 min), adsorption plateaus was observed due to the fewer accessible adsorption sites and more repulsive interactions between the dye molecules on the adsorbent and the bulk phase^51^.

The kinetic behavior of adsorption of MB and CV dyes was investigated by applying three kinetic models in their non-linear forms, known as pseudo-first-order, pseudo-second-order, and intra-particle diffusion models. The pseudo-first-order kinetic and the pseudo-second-order kinetic models are described by Eqs. (9) and (10)^52^ respectively.

9 $${q_t}={q_e}\left( {1 - {e^{ - {k_1}t~}}} \right)$$

10 $${q_t}=\frac{{q_{e}^{2}{k_2}t}}{{1+{k_2}{q_e}t}}$$

Where q_e_ and q_t_ are the adsorbed dyes (mg/g) at equilibrium and at time t (min), respectively. k_1_ (min^−1^), and k_2_ (g/mg·min) are the rate constants of the pseudo-first order and pseudo-second-order kinetic models, respectively.

The fitted plots for the adsorption of MB and CV dyes onto the OP, PC, and ZnO-NR@PC composite are presented in Figs. 9b–d and 10b–d, and their related parameters are in Table 2. The adsorption kinetics of MB and CV dyes began quickly, increased a little, and finally plateaued after 90 min. Based on the higher correlation coefficient (R^2^) and higher adsorption capacity (q_e_), the pseudo-second-order kinetic model displayed the best ability to fit the experimental data of adsorption of MB and CV dyes onto the OP, PC, and ZnO-NR@PC composite. The estimated q_e_ values from the adsorption of MB are 24.58 mg/g (OP), 26.17 mg/g (PC), and 28.79 mg/g (ZnO-NR@PC). The estimated q_e_ from the adsorption of CV are 9.42 mg/g (OP), 24.00 mg/g (PC), and 28.48 mg/g (ZnO-NR@PC). Although the experimentally determined value is higher, the estimated adsorption capacity from kinetic analysis displayed the same order (ZnO-NR@PC > PC > OP).

As the difficulty of determining the rate-controlling step by the pseudo-first order or the second-order models, the Weber-Morris (WM) intra-particle diffusion model was used to investigate whether the process was controlled by film diffusion (i.e., the movement of adsorbate ions from the bulk solution to the external surface of the adsorbent) or intraparticle diffusion (i.e., the movement of adsorbate ions into the interior of the adsorbent)^53^. The non-linear WM model is expressed by Eq. (11).

11 $${q_t}={k_{diff}}{t^{0.5}}+C$$

Where q k_diff_ (mg/g·min^1/2^) is the intra-particle diffusion rate constant, and C (mg/g) is the boundary layer thickness.

As presented in Table 2, the intra-particle diffusion model displayed the least agreement with the experimental data. Thus, the entire adsorption process may be controlled by external mass transfer and intraparticle diffusion. Thus, the pseudo-second-order kinetic model is the best model that fits the experimental results for the adsorption of MB and CV dyes onto the OP, PC, and ZnO-NR@PC materials. This suggests that the chemisorption process, which entails the exchange of electrons between the absorbent and adsorbate, is most likely the rate-limiting step^54^.

**Table 2:** Adsorption kinetic parameters of MB, and CV dyes.

Adsorbent kinetic models									
Adsorbent	Pseudo-first order			Pseudo-second order			Intra-particle diffusion		
	q_e_ (mg/g)	K_1_ (min^−1^)	R^2^	q_e_ (mg/g)	K_2_ (g/mg·min)	R^2^	K_diff_ (mg/g·min^0.5^)	C (mg/g)	R^2^
Parameters of MB adsorption									
OP	21.87	0.097	0.959	24.58	0.005	0.970	1.44	9.410	0.736
PC	22.64	0.119	0.936	26.17	0.009	0.952	0.693	16.49	0.519
ZnO-NR@PC	23.84	0.146	0.972	28.79	0.009	0.975	1.109	14.47	0.661
Parameters of CV adsorption									
OP	8.09	0.066	0.949	9.42	0.008	0.994	0.648	2.28	0.908
PC	21.80	0.114	0.923	24.00	0.007	0.989	1.259	11.05	0.817
ZnO-NR@PC	22.50	0.136	0.830	28.48	0.009	0.970	1.169	12.783	0.868

#### Effect of temperature and thermodynamic analysis

**Fig. 11:**
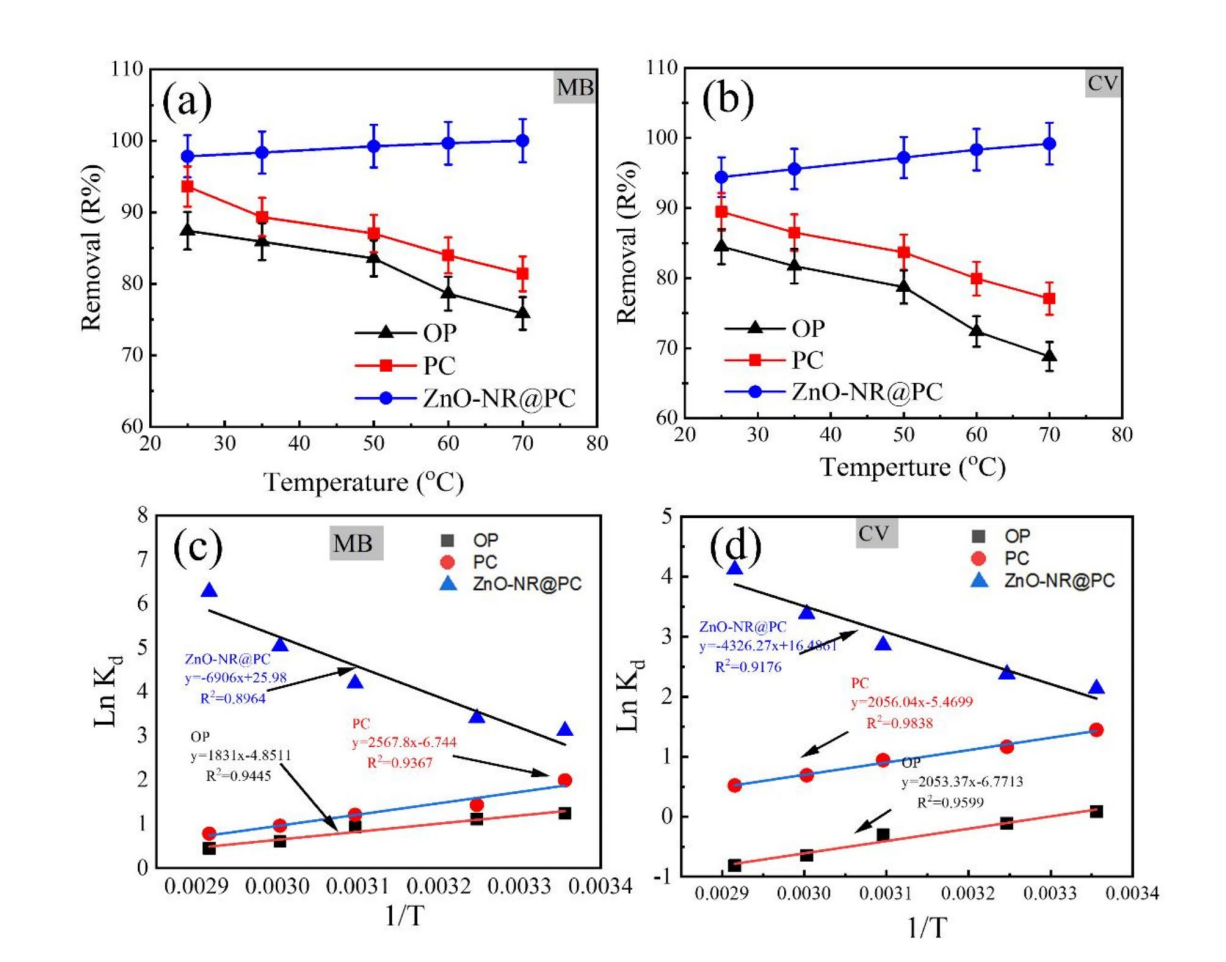
Effect of temperature on the removal of (**a**) MB and (**b**) CV dyes from their aqueous solutions. (**c**,**d**) Van’t Hoff plots of MB and CV adsorption onto the OP, PC, and ZnO-NR@PC composite [M = 0.05 g, t = 90 min, C_0_ = 50 mg/L, pH 10].

The effect of temperature on the removal efficiency of MB and CV dyes was investigated at a temperature ranging from 25 to 70 °C. From Fig. 11a,b, by increasing the reaction temperature, the removal percentage of MB and CV dyes onto the OP and the PC adsorbents were reduced, which indicates an exothermic process. On the other hand, the removal percentage of both dyes by the ZnO-NR@PC composite slightly increased by increasing the temperature, which indicates an endothermic process. The thermodynamic parameters were determined to understand the adsorption behaviors. The equilibrium adsorption experiments were conducted at five distinct temperatures within a range of 25–70 °C. The change in Gibbs free energy ($\Delta {{\text{G}}^{\text{o}}}$), enthalpy ($\Delta {{\text{H}}^{\text{o}}}$), and entropy ($\Delta {{\text{S}}^\circ }$) were estimated using Eqs. (12–14)^55^.

12 $$\ln {K_d}=\frac{{\Delta {S^\circ }}}{R} - \frac{{\Delta {H^\circ }}}{{RT}}~$$

13 $$\Delta {G^0}=\Delta {H^\circ } - T\Delta {S^\circ }~$$

14 $$\Delta {G^0}= - RT\ln {K_{d~}}~$$

Where ΔS° (J/mol. K), ΔG° (kJ/mol), and ΔH° (kJ/mol) represent the changes in entropy, Gibbs free energy, and enthalpy, respectively. T is the adsorption temperature (Kelvin), R is the gas constant (8.3145 J/mol. K), and K_d_ (Q_e_/C_e_) is the change in kinetic energy.

The numerical values of ΔH°, ΔS°, and ΔG° were estimated from the slope and intercept of the linear Van’t Hoff plots of ln K_d_ vs. 1/T (Fig. 11c,d). From Table 3, the negative ΔG^o^ values obtained form the adsorption of MB and CV dyes by the PC, and ZnO-NR@PC composite indicates the adsorption is a spontaneous and thermodynamically favorable process^56^. In addition, the obtained ΔG^o^ values for the adsorption of MB and CV dyes by the PC and ZnO-NR@PC composite in the range of kJ/mol − 20 < ΔG^o^ < 0, which shows that the adsorption is a physical process. On the other hand, the positive ΔG^o^ values obtained from the adsorption of CV dye by the OP indicate the adsorption is not a spontaneous and thermodynamically unfavorable process. For the adsorption of MB and CV onto the ZnO-NR@PC composite, the values of ΔS° and ΔH° are positive. The positive ΔS° value indicates a decrease in randomness at the liquid-solid interface during the adsorption^57^. This suggests that the adsorption process leads to a more ordered arrangement of dye molecules at the interface due to specific interactions between the adsorbate and adsorbent. The positive values of ΔH° denote the endothermic nature of the adsorption process. Suggesting an increase in temperature enhances the adsorption performance as heating the active sites of adsorbents strengthens the bonds between the adsorbate molecules. In contrast, the ΔS° and ΔH° values from adsorption of MB and CV on the OP and PC are negative. The negative ΔS° values indicate an increase in randomness and disorder at the liquid-solid interface during the adsorption, and the negative values of ΔH° indicate that the process is exothermic^58^.

**Table 3:** Thermodynamic parameters for MB and CV adsorption by OP, PC, and ZnO-NR@PC composite.

Adsorbent	T (K)	ΔG° (kJ/mol)	ΔH° (kJ/mol)	ΔS° (kJ/mol. K)
Thermodynamic parameters of MB adsorption				
OP	298	− 3.21	− 15.22	− 0.04
	308	− 2.80		
	323	− 2.20		
	333	− 1.79		
	343	− 1.39		
PC	298	− 3.47	− 21.35	− 0.06
	308	− 2.87		
	323	− 1.97		
	333	− 1.37		
	343	− 0.77		
ZnO-NR@PC	298	− 8.14	57.42	0.22
	308	− 9.24		
	323	− 13.64		
	333	− 15.84		
	343	− 18.04		
Thermodynamic parameters of CV adsorption				
OP	298	0.81	− 17.07	− 0.06
	308	1.41		
	323	2.31		
	333	2.91		
	343	3.51		
PC	298	− 2.2	− 17.10	− 0.05
	308	− 1.7		
	323	− 0.95		
	333	− 0.45		
	343	0.05		
ZnO-NR@PC	298	− 5.75	35.97	0.14
	308	− 7.15		
	323	− 9.25		
	333	− 10.65		
	343	− 12.05		

### The possible adsorption mechanism

**Fig. 12:**
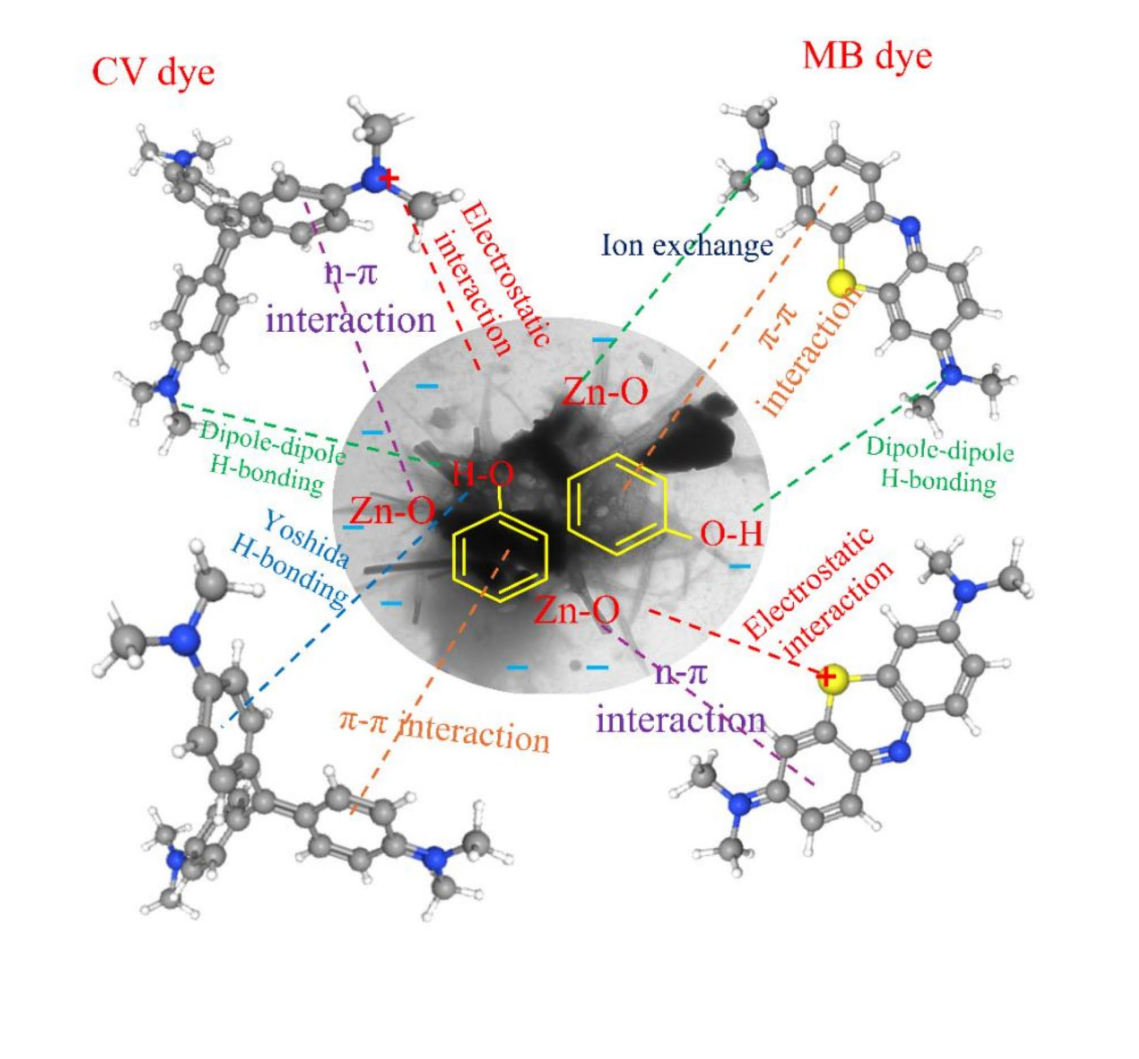
The possible mechanism for adsorption of MR and MO onto ZnO@AC composite.

The possible adsorption mechanism is not only affected by the nature of the adsorbent and adsorbate but also depends on the adsorbate/adsorbent interactions. As discussed in section “Effect of pH”, the adsorption is enhanced by increasing the pH of dye solutions; this means the electrostatic interactions take place during the adsorption process. Additionally, as in section “Characterization of adsorbents derived from waste OP”, the pH_ZPC_ values of the PC and ZnO-NR@PC composite are 6.66 and 8.28, respectively. Thus, at pH > pH_ZPC_, the surface of the adsorbents possesses a negative charges. Thus, the cationic dye molecules (MB and CV) are absorbed on the adsorbent surface due to the electrostatic force between the negative charge of the adsorbent surface and the positive charge of CV dye^32^. As showed in FT-IR, the adsorbent materials displayed a broad band at about 3500 cm^−1^ assigned for OH groups for the adsorbed waster molecules on the surface of the adsorbents. Thus, hydrogen bonding (H-bonding) can formed between the hydrogen of these OH groups (H-donor) with oxygen (O) or nitrogen (N) atoms in the dye molecules (H-acceptor), this kind of H-bonding is known as dipole-dipole H-bonding. Moreover, H-bonding between these OH groups with the aromatic rings in the dye molecules can form, and this type is known as Yoshida H-bonding. The H-bonding can be confirmed by the observable alterations in absorption peaks of the OH groups after adsorption both MB, and CV dyes. The n-π interaction between the oxygen atoms on the ZnO and the π-system in the dye molecules is a possible item participating in the adsorption mechanism^59^. From the FTIR spectrum (Fig. 2d), the small bands for Zn-O, which were located between 650 and 1050 cm^−1^ were remarkably diminished, confirming the presence of n-π interactions. Additionally, the π-π interactions (π^+^–π electron donor–acceptor) between the π-electrons of carbon (e-donor) and the π-electrons of the amine groups (e-acceptor) of the dye molecules can occur. Finally, the ion exchange between the O, N and S atoms of the dye molecules and the Zn ions is a possible way. Hence, the adsorption mechanism of MB and CV dye with the PC and ZnO-NR@PC composite can be controlled by electrostatic attraction, H-bonding, Yoshida H-bonding, n–π interaction, π-π interaction, and ion exchange. Based on the above disscussion, the suggested mechanism is in Fig. 12.

### Regeneration

**Fig. 13:**
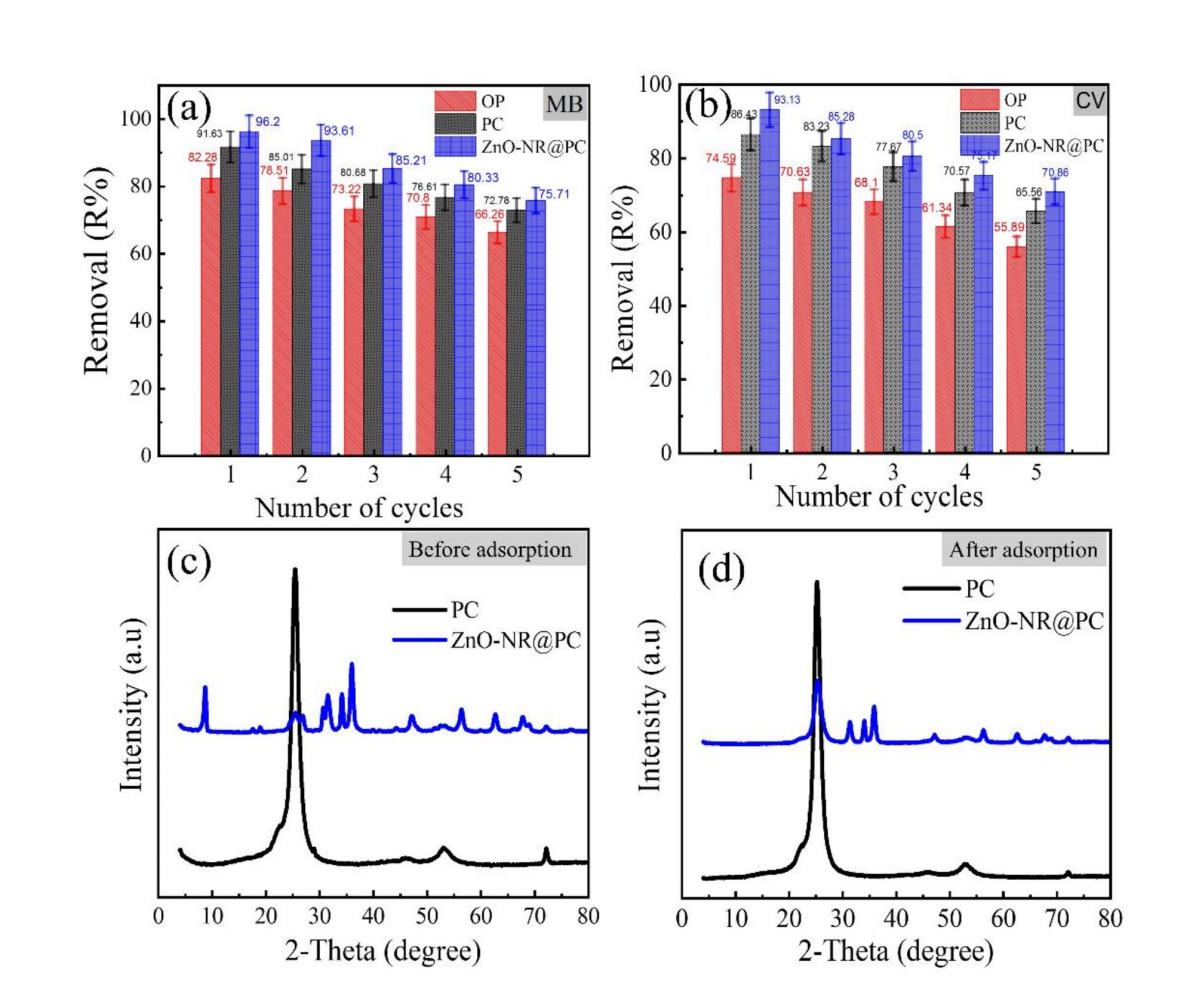
(**a**,**b**) Regeneration of the OP, PC, and ZnO-NR@PC composite up to five successive desorption-adsorption cycles. XRD patterns of the PC and ZnO-NR@PC materials before (**c**) and after (**d**) MB dye adsorption.

To reduce the total cost of the adsorption treatment process, the adsorbent material should be utilized many times without a considerable reduction in its overall adsorption efficieny^60^. In our study, the reusability of the OP, PC, and ZnO-NR@PC composite was examined for five cycles using adsorption/desorption toward MB and CV dyes. The adsorption study was performed at 60 min at room temperature; after each cycle of adsorption, the remaining concentrations of MB, and CV dyes in the residual filtrate were recorded. Then, the regeneration studies were washing the OP, PC, and ZnO-NR@PC composite loaded with adsorbed dyes twice with ethanol and distillated water for 60 min. After that, the regenerated adsorbents were reused for MB and CV dye adsorption, and five cycles of regeneration and adsorption were carried out in succession. The corresponding percentages of dye removal in Fig. 13a,b display that the OP, PC, and ZnO-NR@PC composite depict good recyclability after five cycles of desorption-adsorption. As shown in Fig. 13a, the removal percentages of MB by OP, PC, and ZnO-NR@PC composite decreased by 19.5, 20.57, and 21.30%, respectively. Moreover, in Fig. 13b, the removal percentages of CV onto the OP, PC and ZnO-NR@PC composite were reduced by 25.07, 26.46 and 23.91%, respectively. Thus, the ZnO-NR@PC composite displayed highly reusable properties for the removal of cationic dyes.

### Chemical stability of the adsorbents

After multiple adsorption-desorption cycles, the chemical stability of the PC and ZnO-NR@PC materials derived from waste OP was investigated by XRD and SEM analysis. The XRD patterns of the PC and ZnO-NR@PC materials before (Fig. 13c), and after (Fig. 13d) MB dye adsorption are the same without any noticeable changes. In addition, the SEM images of the PC and ZnO-NR@PC materials before and after MB dye adsorption in Fig. 4 display similar structres. Thus, combining the reusability of adsorption, XRD in Fig. 13c,d, and SEM images in Fig. 4 indicates that our prepared adsorbent materials are chemically stable during the adsorption process. This further confirms the physical adsorption process.

### The applicability of the adsorbents toward real water samples

**Fig. 14:**
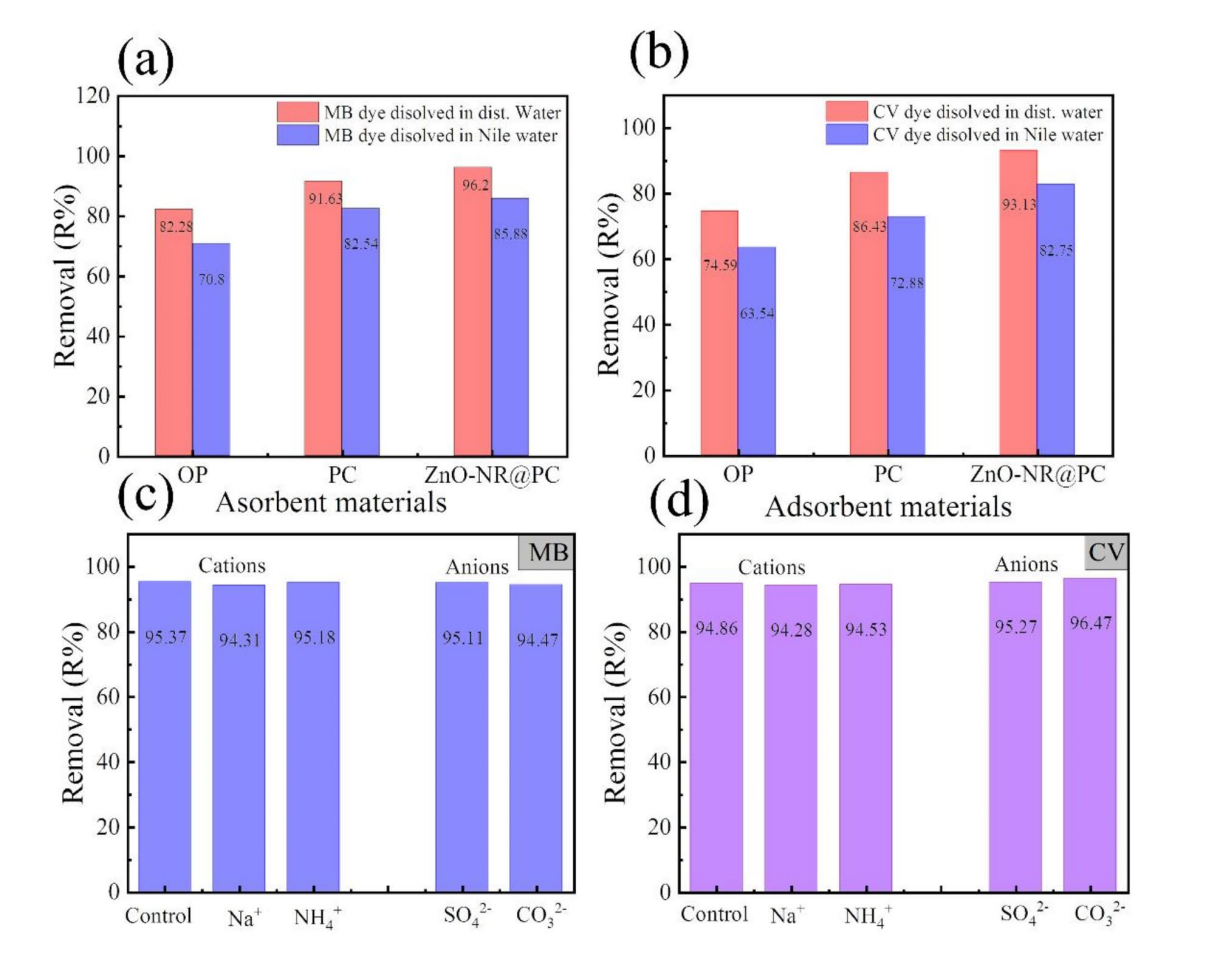
Effect of OP, PC, and ZnO-NR@PC composite toward (**a**) MB and (**b**) CV prepared by distillated water and Nile River water samples. Effect of coexisting cations and anions on (**c**) MB and (**d**) CV adsorption by ZnO-NR@PC composite.

The effect of utilizing real water as dye solvent on the overall adsorption process was investigated. Here we utilize Nile water with a condition presented in our previous work^61^. In our experimental work, we compare the removal efficiency of the PC and ZnO-NR@PC composite toward MB and CV dyes dissolved with distillated water and Nile waster at the same conditions. The adsorption experiment was performed by mixing 30 mL of dye solution with a in a concentration of 50 mg/L with 0.03 g ZnO-NR@PC composite, each solution was stirred for 90 min. After that, it was subjected to centrifugation, and the remaining dye concentrations were determined by a UV-vis spectrophotometer at wavelengths of 664 and 590 nm for MB and CV dye, respectively. The removal percentages in Fig. 14a,b showed that the removal percentages of MB and CV from the real sample displayed not much difference with those obtained by dyes in distillated water.

### Effect of coexistence cations and anions on dye removal

The real wastewater contaminated with dyes contains various inorganic salts that affect the overall adsorption efficiency; thus, it is important to investigate the effect of such coexisting ions. As illustrated in Fig. 14c, the co-existing cations had a minor impact on the adsorption of MB, while the anions displayed quite a small affect on MB dye. The small reduction in MB in the presence of cations can be attributed to the competitive effect between the cations and the positively charged MB dye molecules on the sites available for the sorption process; thus, a reduction in adsorption efficiency will occur^62^. On the other hand, the effect of ions on the CV dye adsorption (Fig. 14d) showed that the presence of cations leads to a little reduction in the removal efficiency, while the anions displayed a positive effect as indicated by enhancing the removal efficiency. The increase in the adsorption of CV in the presence of anions could be attributed to the decrease in the intermolecular forces between the CV dye molecules^63^.

## Comparison of our adsorbent with others

The overall adsorption capacity is mainly influenced by the nature of the adsorbent, the primary source of the adsorbent, the adsorption conditions, and the type of the adsorbent. Thus, great efforts have been devoted to finding or developing effective adsorbent materials with high adsorption efficiency, eco-friendliness, as well as low cost. Thus, in this section, our absorbent materials toward MB and CV dyes were compared with other adsorbents in the literature as listed in Table 4. The survey data showed that our adsorbents displayed competitive adsorption ability towards MB and CV dyes. In addition, our adsorbents are cost-effective, easy to prepare, and with good reusability. All these advantages are encouraging for practical applications for the treatment of effluents containing dyes, such as textile industry effluents.

**Table 4:** Adsorption of MB and CV dyes by our adsorbents and various adsorbents derived from biowaste materials.

Adsorbent	Dye	q_m_ (mg/g)	References
Porous carbon from hemp seeds	MB	49.89	^64^
AC from fir bark	MB	330	^65^
Biochare from wheat straw	MB	62.5	^66^
Hydrochar-derived porous carbons	MB	833.33	^67^
Magnetite nanoparticles loaded Fig leaves (MNLFL) and loaded Azolla (MNLA)	MB	61.72 and 25	^68^
Barley bran (BB) and enset (Ensete ventricosum midrib leaf (EVML)	MB	63.2 and 35.5	^69^
Carbon microspheres (CMs) from date palm	MB	409.84	^70^
Carbon from cotton stalk (CS)	MB	198	^71^
ZnO-NR@PC-derived from orange peel	MB	74.45	This work
Activated carbon prepared from lemon wood (ACL)/Fe3O4 magnetic nanocomposite	CV	35.3	^72^
AC-derived from Leftover noodles/ZnO (LNAC-ZnO)	CV	79.8	^73^
Magnetite nanoparticles loaded Fig leaves (MNLFL) and loaded Azolla (MNLA)	CV	53.47 and 30.21	^68^
Carbonaceous material from waste cotton and polyester	CV	114	^21^
NaOH-activated Aerva javanica leaf (NAJL)	CV	315.2	^74^
ZnO-NR@PC-derived from orange peel	CV	74.89	This work

## Conclusions

In summary, both MB and CV cationic dyes have been removed from aqueous solution by batch adsorption technique. In our study, orange peel has been utilized to prepare the PC and the ZnO-NR@PC composite adsorbents. The adsorptive properties of the prepared materials displayed high affinity toward MB and CV dyes at basic conditions. The experimental data of adsorption MB and CV dyes are fitted with the pseudo-second order-kinetic model. In addition, the adsorption of MB and CV onto the PC and the ZnO-NR@PC composite are fitted with the Langmuir isotherm model, and the estimated maximum adsorption capacity of the ZnO-NR@PC composite toward MB and CV dyes is 74.45 and 74.89 mg/g, respectively, which are higher than those achieved by OP and PC materials. The thermodynamic analysis confirmed that the adsorption process is a spontaneous and thermally favorable process. In addition, our adsorbent materials displayed high stable adsorption performance after five cycles. Thus, as future perspectives of this study, it is recommended to utilize orange peel as a precursor for preparing carbon-based adsorbents with various morphologies for the recovery of various organic pollutants such as dyes, phenols, and pharmaceutical compounds from aqueous solutions and industrial wastewaters.

## Data Availability

The authors confirm that the data supporting the findings of this study are available within the article.
